# Effect of processing methods on fatty acid composition and flavour profile of clarified butter (ghee) obtained from Deoni and Holstein Friesian cow breeds

**DOI:** 10.1016/j.fochx.2025.102489

**Published:** 2025-04-22

**Authors:** Deepshikha Kataria, Gurmeet Singh

**Affiliations:** The University of Trans-Disciplinary Health Sciences and Technology, Bengaluru 560064, India

**Keywords:** Clarified butter, Fatty acid, Volatile compounds, *Deoni*, *Holstein Friesian*

## Abstract

The study investigated the impact of three processes—curd-butter (CD), cream-butter (CM) and fermented cream-butter (FC) on fatty acid composition and volatile profiles of clarified butter (ghee) obtained from two cow breeds, Deoni (DN) and Holstein Friesian (HF). Gas chromatography-mass spectrometry (GC–MS) identified 26 fatty acids in ghee. There was no difference in fatty acid composition due to processing. Significant differences were observed by breed, with HF ghee exhibiting higher polyunsaturated fatty acids and α-linolenic acid than DN ghee. Headspace GC–MS detected 57 volatile compounds. Ghee made with CD and FC processes exhibited higher levels of acids, alcohols, heat degradation products, lactones, ketones, 5-hydroxymethylfurfural and maltol in both breeds. These findings suggest that processing methods alter ghee's volatile compounds without significantly affecting fatty acid composition. Since ghee is a complex lipid, untargeted lipidomics and metabolomics studies can provide further insight into the effects of processing on ghee. This study can guide dairy processors in selecting milk sources and ghee processes to enhance flavour in ghee while maintaining fatty acid composition across cow breeds for product improvement.

## Highlights

• The study compared the fatty acid and volatile composition of clarified butter (ghee) obtained from two cow breeds using three processing methods.
• Processing methods had no significant impact on the fatty acid composition of ghee, while differences between the breeds were observed.
• Ripened ghee showed higher acids, alcohols, thermal degradation products, lactones, ketones, 5-Hydroxymethylfurfural and maltol.
• This study highlights the role of milk source and processing methods in shaping nutritional and sensory attributes of ghee.

## 1 Introduction

Ghee, also known as clarified butter, is gaining popularity in other parts of the world. In Ayurveda, the traditional Indian knowledge system, ghee is valued as a nutritious and therapeutic food (Achaya, 2018). The culinary, nutritional and therapeutic values of ghee are widely believed to be dependent on the source of milk and the processing method used to obtain ghee (Dorni et al., 2018; Erfani et al., 2019; Kataria & Singh, 2024; Sserunjogi et al., 1998; Sulieman et al., 2013). The most frequently referred source of milk in Ayurvedic literature is the cow and the most frequently referred processing method is the curd-butter ghee method (Kataria & Singh, 2024). Over the last few decades, as India has undergone a revolution in milk and dairy processing, the breeds of cows and industrial ghee processing methods have evolved compared to those used in traditional practices. Most cow milk today comes from hybrid or crossbred cows. The industrial method of ghee manufacturing uses the cream-butter and direct cream methods (Kwak et al., 2013; Ray, 2020).

Despite the high consumption of clarified butter (ghee), only a few studies have been conducted on the effect of processing on the fatty acid composition and volatile compounds in ghee (Supplementary Table S1). While within-study comparisons are informative, comparisons across these studies have been of limited use as the gas chromatography protocols, ghee processing methods, and milk sources have varied from study to study. As a result, findings from different studies have been many times inconsistent with each other (Bhide, 2014; Joshi, 2014; Tyagi et al., 2008). Even where statistically significant differences in fatty acids have been reported, the magnitude of variations has been small on an absolute level and appears insufficient to confer any nutritional or therapeutic implications. The findings from select studies are summarized in supplementary table S2.

Similar challenges of interpretation occur between different studies on volatile compounds of ghee. While Yadav and Srinivasan (1984) reported that ripening cream produced higher free fatty acids and carbonyls, Bhide (2014) found that direct-cream ghee was more flavourful in sensory analysis. The number of compounds reported in the various studies also differed significantly, from 16 to 36 (Wadodkar et al., 2002).

This study was designed to address the limitations in comparing the fatty acid composition and volatile profiles of ghee across different processing methods, which arise due to variations in analytical methodologies and milk sources across different publications. Three processes were compared in this study – two ripened ghee processes and one unripened ghee process. The ripened ghee processes were curd-butter (CD) and fermented cream-butter (FC) methods, while the unripened process was the cream-butter (CM) method. Further, milk from two cow breeds, the native Deoni (DN) cow and the crossbred Holstein Friesian (HF) cow, was used to evaluate the consistency of the effects of processing on fatty acid composition and the volatile profile of ghee across milk sources. The 2 × 3 experimental design resulted in six ghee samples which were evaluated for their fatty acid composition and volatile profile (Table 1). This experimental design enabled an understanding of whether the observed molecular changes in fatty acids and volatile compounds resulting from processing were specific to milk derived from a single breed or exhibited a more generalized pattern across different breeds.

**Table 1:** Ghee processing methods and cow breeds used in the study with respective sample codes.

Cow breed	Curd-butter method (CD)	Fermented cream-butter method (FC)	Cream-butter method (CM)
	Ripened	Ripened	Unripened
Deoni (DN)- Native breed	DN CD	DN FC	DN CM
Holstein Friesian (HF) - Hybrid	HF CD	HF FC	HF CM

Note: The abbreviations used in this table are also used in the methods and results 2, 3.

Ghee has a fatty acid and flavour profile distinct from all other edible fats and oils. These distinct characteristics include short-chain fatty acids (SCFA), odd-chain fatty acids and conjugated linoleic acids (CLA). Some of these characteristics are being studied for their effect on human physiology, such as the anti-inflammatory effect of butyric acid, a SCFA (Saini & Keum, 2018; Simopoulos, 2002), cell membrane stabilizing effect of pentadecanoic acid, an odd chain fatty acid (Dąbrowski & Konopka, 2022; Venn-Watson et al., 2020), and the cardio-protective effect of CLA (Benjamin & Spener, 2009; Collomb et al., 2006; Silveira et al., 2007). A scheme was developed to compare the six ghee samples to capture the effect of processing on these individual fatty acids and fatty acid groupings. This scheme involved comparing the ghee at two class-level clusters, four special group-level clusters, and individual fatty acids. The two class-level clusters were chain-length and degree of saturation class-level clusters. The four specialty fatty acid clusters were odd-chain fatty acids, CLA, omega-3 (ω-3) and omega-6 (ω-6) fatty acids. These fatty acid class levels and groupings are given in Table 3 in the 3.1 section. Similarly, the volatile compounds in ghee were also compared based on their functional group classification at both the overall and the individual compound levels. This comparison is presented in Table 7 of Section 3.2.

## 2 Materials and methods

### 2.1 Sample collection and ghee processing

The study collected raw, fresh milk from two cow breeds, DN and HF, from the Experimental Dairy, Southern Regional Station of the Indian Council of Agricultural Research-National Dairy Research Institute, Bengaluru, Karnataka, India. Samples were collected within 1–2 h of milking in the morning to ensure uniformity and then pasteurized in a locally fabricated stainless-steel double-jacketed steam vessel (SLV Engineering, Bengaluru, Karnataka, India), followed by cooling for further processing.

To produce ghee, the milk from each cow breed was divided into three equal parts from the same lot and processed using CM, CD and FC processes. The ghee processing methods used in this study were adapted from the literature (Battula et al., 2020; Ganguli & Jain, 1973; Kumbhare et al., 2021) with some modifications.

A centrifugal cream separator was used to extract cream from milk for the CM process. The extracted cream was churned to make butter using an electric blender. This process did not involve ripening or fermentation of the cream with a starter culture and is referred to as the unripened CM process.

The two ripened ghee, CB and FC, were prepared as described below. For the CB process, the milk was inoculated with a 2 % starter culture of *Streptococcus lactis sub* sp. *diacetylactis*, obtained from the National Collection of Dairy Cultures, National Dairy Research Institute, Karnal, Haryana, India. The cultured milk was incubated at 30 ± 5 °C for 22 h until the curd was set due to ripening. The curd was churned using an electric blender to make curd butter.

The FC process involves the extraction of cream using a centrifugal cream separator. The cream was inoculated with a 2 % starter culture of *Streptococcus lactis sub* sp. *diacetylactis* and incubated at 30 ± 5 °C for 22 h till ripening or fermentation of the cream. The fermented cream was churned using an electric blender to make fermented cream butter.

The butter obtained from the above-mentioned processes was clarified individually to make CM, CD, and FC ghee. The butter was clarified or heated in a stainless-steel pan on the direct flame until the temperature reached 120 °C and the moisture content reduced to <0.5 %. The ghee thus prepared was cooled, filtered and packed in air-tight glass containers for further analysis. Table 1 shows the samples generated in the study.

### 2.2 Analysis of fatty acids in ghee by GC–MS

#### 2.2.1 Esterification of fatty acids

Lipids in ghee were converted to Fatty Acid Methyl Esters (FAMEs) as described by Simionato et al. (2010) with some modifications. To a screw-cap tube containing approximately 40–60 mg of ghee, 5.0 mL of 0.25 mol L^−1^ sodium methoxide (Spectrochem Pvt. Ltd., Mumbai, India) in 1:1 mixture of methanol (Sigma-Aldrich, Darmstadt, Germany) and diethyl ether was added. The tube was agitated vigorously for 3 min, and the contents were transferred quantitatively to a separating funnel with 6 mL of hexane (Sigma-Aldrich, Darmstadt, Germany). Next, 15 mL of saturated sodium chloride solution was added, and the mixture was agitated again. The contents were rested until phase separation. The hexane layer containing esterified fatty acids was collected over sodium sulphate and filtered through a 0.22 μm nylon filter into a glass vial for analysis (ISO 12966-2, 2017).

#### 2.2.2 Analysis of FAMEs

The FAMEs were analyzed by GC–MS using a Shimadzu model Nexis 2030 series (Shimadzu, Japan) equipped with a fused silica capillary column (100 m length, 0.25 mm i.d., 0.2 μm df, 100 % bonded biscyanopropyl polysiloxane, Rt-2560, Restek, USA). The injection temperature was set at 240 °C. Helium was used as the carrier gas at a 1 mL/min (min) flow rate. The column temperature was programmed at 100 °C for 4 min, followed by a ramp of 5 °C/min, reaching up to 190 °C with a holding time of 5 min. A second ramp of 2 °C/min was set and raised to 220 °C, followed by a hold time of 16 min. Mass spectrometry analysis was performed using a GCMS-TQ8040 NX mass spectrometer (Shimadzu, Japan). The ion source temperature was set at 230 °C in the detector, with an interface temperature of 220 °C. The FAMEs were identified by comparing the retention times of the derivatized fatty acids with those in the Supelco standard mix (47,885, 37-FAME mix standard, Sigma-Aldrich, USA).

### 2.3 Analysis of volatile compounds in ghee by headspace GC–MS

#### 2.3.1 Extraction of volatile compounds from ghee

Methanol was used to extract volatile compounds from ghee following the method of Obi et al. (2018) with some modifications. Ghee (0.5–0.6 g) was extracted with 2 mL methanol in a 25 ml centrifuge tube. The contents were homogenized for 1 min using a vortex, followed by centrifugation for 4 min at 4000*g* at 4 °C. After centrifugation, the clear supernatant was collected in another centrifuge tube and stored at −80 °C for 5 min to solidify triglycerides. This tube was again centrifuged for 4 min at 4000*g* at 4 °C to separate the solidified triglycerides. An aliquot of 1 mL clear supernatant was collected in a headspace vial. The collected supernatant solvent was evaporated under a gentle stream of nitrogen. The vial was crimped tightly with a 20 mm aluminum cap and kept in the headspace autosampler of the GC–MS for analysis.

#### 2.3.2 Analysis and quantification of volatile compounds in ghee

Volatile compounds in ghee were analyzed by GC–MS (*Nexis* GC-2030 series, Shimadzu, Japan) coupled with automatic static headspace (HS) sampler HS-20 and MS detector (TQ-8040NX, Shimadzu, Japan). The column used was Rtx-Volatiles, featuring a cross-bond diphenyl/dimethyl polysiloxane stationary phase, 30 m in length, 0.25 mm in internal diameter, and 1 μm in film thickness. The oven temperature in the gas chromatograph was set at 80 °C with a sample and transfer line temperature of 150 °C. The GC program was initialized at 45 °C with first ramp of 150 °C at the rate of 5 °C/ min and a second ramp of 250 °C at the rate of 5 °C/min with 5 min hold time. The ion source temperature in MS was set at 220 °C with 250 °C interface temperature. The detector voltage was 70 eV with an ion scan range of 45–500 *m*/*z*. Nine volatile compound standards (maltol, 5-hydroxymethylfurfural, 2-tridecanone, 2-pentadecanone, 2-undecanone, δ-octalactone, δ-decalactone, γ-dodecalactone and δ-dodecalactone) which were purchased from Supelco, Sigma-Aldrich, Germany, Europe, were used for quantification. The linear curve equations of these standards have been provided in supplementary table S3. The related cromatograms and linear curves can be acessed from Supplementary material 2.

### 2.4 Statistical analysis

Three samples were prepared for fatty acid analysis and each measurement was conducted in triplicate. For volatile compounds, one sample of each type of ghee was analyzed in duplicate. *t*-test was conducted using the Statistical Package for the Social Sciences (SPSS) version 22.0 for Windows (IBM SPSS Inc., Chicago, USA). Two-way analysis of variance (ANOVA) was performed using statistical software developed by Assaad et al. (2015). All data were presented as mean ± SEM. Tukey's post-hoc test was used to derive statistical significance at 5 % level (*p* < 0.05).

## 3 Results and discussion

### 3.1 Fatty acid profile of ghee

A total of 26 fatty acids were identified in the 6 ghee samples using the 37 FAME mix standard. The fatty acids included in the various classes are listed in Table 2, Table 3, along with their respective compositions. In terms of fatty acid class level clusters based on chain length, there were 2 SCFA, 4 medium-chain fatty acids (MCFA) and 20 long-chain fatty acids (LCFA). In terms of the degree of saturation, there were 14 saturated fatty acids (SFA), 6 monounsaturated fatty acids (MUFA) and 6 polyunsaturated fatty acids (PUFA), including linoleic acid (cis-9,12-Octadecadienoic acid, C18:2 n-6c) (LA) and α-linolenic acid (cis- 9,12,15-octadecatrienoic, C18:3, n-3) (ALA). The SFA was 65 to 70 %, MUFA was 20 to 25 %, and PUFA was 3 to 4 % in all the ghee samples (Table 3, Table 4). The most abundant long-chain SFA in ghee was palmitic acid (C16:0), followed by stearic acid (C18:0) and myristic acid (C14:0). Oleic acid (18,1) was the most abundant long-chain MUFA in ghee. 83–85 % of the fatty acids were LCFA, 7 to 9 % were MCFA and 6 to 7 % were SCFA (Table 2). Out of the 26 fatty acids, 21 were even-chain, while 5 were odd-chain fatty acids (C11:0, C13:0, C15:0, C17:0, and C17:1). Odd-chain fatty acids make up approximately 2 % of ghee, with pentadecanoic acid (C15:0) being the most abundant odd-chain fatty acid. The results of our study align with those reported in previous literature on ghee (Kwak et al., 2013; Longvah et al., 2017).

**Table 2:** Fatty acid (FA) composition of DN and HF ghee made with three processing methods (FA mg/g ghee)⁎

Sr. No.	Fatty acids	Short name	Degree of Saturation	Chain length	DN			HF		
					CD	CM	FC	CD	CM	FC
**1**	Butyric acid ME	C4:0	SFA	SCFA	39.2 ± 0.81	38.9± 1.18	37.6 ± 0.85	40.7 ± 0.15	44.2 ± 2.13	42.2 ± 1.54
**2**	Caproic acid ME	C6:0	SFA	SCFA	25.0 ± 0.58	23.9 ± 0.60	23.4 ± 0.56	24.1 ± 0.33	25.9 ± 0.93	24.6 ± 1.1
**3**	Caprylic acid ME	C8:0	SFA	MCFA	14.4 ± 0.34	13.8 ± 0.37	13.6 ± 0.43	14.1 ± 0.32	14.7 ± 0.65	13.8 ± 0.74
**4**	Capric acid	C10:0	SFA	MCFA	30.1 ± 0.53	28.6 ± 0.85	29.1 ± 0.90	30.4 ± 0.70	30.9 ± 1.50	29 ± 1.46
**5**	Undecanoic acid	C11:0	SFA	MCFA	0.59 ± 0.03	0.55 ± 0.03	0.55 ± 0.05	0.62 ± 0.06	0.63 ± 0.03	0.60 ± 0.03
**6**	Lauric acid	C12:0	SFA	MCFA	34.4 ± 1.08	32.7 ± 0.29	33.4 ± 1.48	38.1 ± 0.78	38.1 ± 1.46	36.4 ± 1.31
**7**	Tridecanoic acid	C13:0	SFA	LCFA	1.33 ± 0.01	1.25 ± 0.06	1.25 ± 0.06	1.25 ± 0.09	1.28 ± 0.06	1.22 ± 0.02
**8**	Myristic acid	C14:0	SFA	LCFA	110 ± 0.44	110 ± 1.38	111 ± 1.87	110 ± 0.67	112 ± 1.47	110 ± 2.14
**9**	Myristoleic acid	C14:1	MUFA	LCFA	10.6 ± 0.49	9.44 ± 0.40	9.97 ± 0.38	9.02 ± 0.36	9.28 ± 0.15	9.39 ± 0.14
**10**	Pentadecanoic acid	C15:0	SFA	LCFA	10.8 ± 0.04	11.3 ± 0.23	11.0 ± 0.12	10.4 ± 0.74	10.1 ± 0.44	10.1 ± 0.51
**11**	Palmitic acid	C16:0	SFA	LCFA	269 ± 2.61	275 ± 1.18	275 ± 0.71	264 ± 5.07	263 ± 4.63	264 ± 6.97
**12**	Palmitoleic acid	C16:1	MUFA	LCFA	13.9 ± 0.47	13.2 ± 0.43	13.3 ± 0.23	12.3 ± 0.63	12.1 ± 0.62	12.6 ± 0.43
**13**	Heptadecanoic acid	C17:0	SFA	LCFA	5.13 ± 0.11	5.36 ± 0.05	5.3 ± 0.03	5.16 ± 0.36	4.92 ± 0.22	4.97 ± 0.23
**14**	Heptadecenoic acid	C17:1	MUFA	LCFA	2.84 ± 0.13	2.77 ± 0.40	2.64 ± 0.14	2.61 ± 0.60	2.76 ± 0.11	2.51 ± 0.22
**15**	Stearic acid	C18:0	SFA	LCFA	115 ± 5.36	127 ± 5.41	126 ± 5.59	121 ± 3.99	120 ± 4.06	122 ± 1.37
**16**	Elaidic acid	C18:1-n9t	MUFA	LCFA	23.2 ± 0.826	19.6 ± 0.24	21.8 ± 0.66	16.9 ± 2.71	16.0 ± 2.11	16.1 ± 2.64
**17**	Oleic acid	C18:1-n9c	MUFA	LCFA	188 ± 3.49	184 ± 6.03	183 ± 3.57	189 ± 5.2	183 ± 5.14	190 ± 5.07
**18**	Linoleic acid (LA)	C18:2-n6	PUFA	LCFA	12.6 ± 0.51	12.2 ± 0.67	12.0 ± 0.3	17.6 ± 1.53	17.0 ± 1.43	17.6 ± 1.47
**19**	Arachidic acid	C20:0	SFA	LCFA	6.09 ± 0.32	6.12 ± 0.19	5.96 ± 0.27	5.98 ± 0.23	6.16 ± 0.34	6.24 ± 0.33
**20**	Gamma-Linolenic acid	C18:3-n6	PUFA	LCFA	2.03 ± 0.07	1.84 ± 0.24	1.71 ± 0.17	1.91 ± 0.12	2.03 ± 0.19	2.04 ± 0.25
**21**	Eicosenic acid	C20:1n9,11c	MUFA	LCFA	3.66 ± 0.22	3.38 ± 0.21	3.1 ± 0.16	3.39 ± 0.24	3.53 ± 0.51	3.67 ± 0.27
**22**	α-Linolenic acid (ALA) ω-3	C18:3-n3	PUFA	LCFA	3.23 ± 0.08	3.12 ± 0.18	2.95 ± 0.15	4.18 ± 0.19	4.15 ± 0.29	4.25 ± 0.11
**23**	Behenic acid	C22:0	SFA	LCFA	5.29 ± 0.21	4.67 ± 0.58	4.7 ± 0.39	5.12 ± 0.34	5.31 ± 0.54	5.4 ± 0.45
**24**	Eicostrienoic acid	C22:3-n6 cis-8,11,14-	PUFA	LCFA	2.22 ± 0.06	2.04 ± 0.25	1.93 ± 0.12	2.28 ± 0.09	2.28 ± 0.17	2.36 ± 0.24
**25**	CLA (c-9, t-11)	C18:2	PUFA	LCFA	8.81 ± 0.40	8.5 ± 0.54	8.61 ± 0.31	7.6 ± 0.25	7.35 ± 0.23	7.73 ± 0.35
**26**	Arachidonic acid	C20:4-n6, (cis-5,8,11,14)	PUFA	LCFA	3.47 ± 0.13	2.96 ± 0.33	2.92 ± 0.24	3.38 ± 0.23	3.48 ± 0.18	3.53 ± 0.27
**Sum of fatty acids**					942 ± 0.09	942 ± 0.22	942 ± 0.16	942 ± 0.05	941 ± 0.31	942 ± 0.28

Abbreviations DN=Deoni, HF=Holstein friesian, CD=Curd-butter ghee

CM = Cream-butter ghee, FC=Fermented cream-butter ghee.

SCFA = Short Chain Fatty Acids; MCFA = Medium Chain Fatty Acids; LCFA = Long Chain Fatty Acids; SFA = Saturated Fatty Acids; USFA = Unsaturated Fatty Acids; PUFA = Polyunsaturated Fatty Acids.

⁎ Values are mean ± SEM of triplicates of three samples.

**Table 3:** Comparison of fatty acid (FA) levels between ripened and unripened processing methods in ghee of *DN* cow breed (FA mg/g ghee)⁎

Fatty acid groups	DN Curd-butter	DN Cream-butter	DN Fermented Cream-butter	DN Cream-butter
Chain Length Classes				
SCFA	64.1 ± 0.88	62.7 ± 1.78	61.0 ± 1.32	62.7 ± 1.78
MCFA	79.5 ± 1.94	75.6 ± 1.45	76.7 ± 2.54	75.6 ± 1.45
LCFA	798 ± 2.4	804 ± 3.44	794 ± 2.06	804 ± 3.44
Degree of Saturation Classes				
SFA	667 ± 6.0	679 ± 8.8	671 ± 3.69	679 ± 8.8
MUFA	242 ± 5.09	232 ± 7.13	233 ± 2.96	232 ± 7.13
PUFA	32.4 ± 0.85^b^	30.9 ± 1.84	30.5 ± 1.07^b^	30.9 ± 1.84
Individual fatty acids				
Butyric acid (SCFA)	39.2 ± 0.81	38.9 ± 1.18	37.6 ± 0.85	38.9 ± 1.18
C15:0 (Odd-chain fatty acid)	10.8 ± 0.04	11.3 ± 0.23	11.0 ± 0.12	11.3 ± 0.23
α-Linolenic acid (ALA) ω-3	3.23 ± 0.08	3.12 ± 0.18	2.95 ± 0.15	3.12 ± 0.18
Conjugated fatty acid (CLA) (c-9, t-11)	8.81 ± 0.40	8.5 ± 0.54	8.61 ± 0.31	8.5 ± 0.54
Specialty groupings				
Odd-chain fatty acids (C11:0, C13:0, C15:0, C17:0, C17:1)	20.69 ± 0.29	21.18 ± 0.3	20.72 ± 0.16	21.18 ± 0.3
ω-3	3.23 ± 0.08	3.12 ± 0.18	2.95 ± 0.15	3.12 ± 0.18
ω-6	20.3 ± 0.56	19.3 ± 1.16	19 ± 0.82	19.3 ± 1.16
Ratio ω-6/ ω-3	6.28	6.18	6.44	6.18

Abbreviations, DN=Deoni, HF=Holstein friesian, CD=Curd-butter ghee, CM = Cream-butter ghee, FC=Fermented cream-butter ghee.

SCFA = Short Chain Fatty Acids; MCFA = Medium Chain Fatty Acids; LCFA = Long Chain Fatty Acids; SFA=Saturated Fatty Acids; USFA = Unsaturated Fatty Acids; PUFA = Polyunsaturated Fatty Acids.

⁎ Values are mean ± SEM of triplicates of three samples.

**Table 4:** Comparison of fatty acid (FA) levels between ripened and unripened processing methods in ghee HF cow breed (FA mg/g ghee)⁎

Fatty acid groups	HF Curd-butter	HF Cream-butter	HF Fermented Cream-butter	HF Cream-butter
Chain Length Classes				
SCFA	64.8 ± 0.47	70.1 ± 2.94	66.8 ± 2.64	70.1 ± 2.94
MCFA	83.2 ± 1.61	84.3 ± 3.53	79.8 ± 3.43	84.3 ± 3.53
LCFA	794 ± 2.06	787 ± 5.1	795 ± 6.28	787 ± 5.1
Degree of Saturation Classes				
SFA	671 ± 3.69	677 ± 4.31	670.0 ± 3.0	677 ± 4.31
MUFA	233 ± 2.96	227 ± 3.71	234 ± 2.39	227 ± 3.71
PUFA	37.7 ± 1.27	36.9 ± 1.01	37.5 ± 1.73	36.9 ± 1.01
Individual fatty acids				
Butyric acid (SCFA)	40.7 ± 0.15	44.2 ± 2.13	42.2 ± 1.54	44.2 ± 2.13
C15:0 (Odd-chain fatty acid)	10.4 ± 0.74	10.1 ± 0.44	10.1 ± 0.51	10.1 ± 0.44
α-Linolenic acid (ALA) ω-3	4.18 ± 0.19	4.15 ± 0.29	4.25 ± 0.11	4.15 ± 0.29
Conjugated fatty acid (CLA) (c-9, t-11)	7.6 ± 0.25	7.35 ± 0.23	7.73 ± 0.35	7.35 ± 0.23
Specialty groupings				
Odd-chain fatty acids (C11:0, C13:0, C15:0, C17:0, C17:1)	20.01 ± 2.58	19.67 ± 1.19	19.42 ± 1.27	19.67 ± 1.19
ω-3	4.18 ± 0.19	4.15 ± 0.29	4.25 ± 0.11	4.15 ± 0.29
ω-6	26.0 ± 1.37	25.4 ± 1.2	25.5 ± 1.69	25.4 ± 1.2
Ratio ω-6/ ω-3	6.22	6.12	6.0	6.12

Abbreviations DN=Deoni, HF=Holstein friesian, CD=Curd-butter ghee, CM = Cream-butter ghee, FC=Fermented cream-butter ghee.

SCFA = Short Chain Fatty Acids; MCFA = Medium Chain Fatty Acids; LCFA = Long Chain Fatty Acids; SFA = Saturated Fatty Acids; USFA = Unsaturated Fatty Acids; PUFA = Polyunsaturated Fatty Acids.

⁎ Values are mean ± SEM of triplicates of three samples.

#### 3.1.1 Effect of processing methods on the fatty acid profile of ghee

A statistical comparison of fatty acid levels between ripened CD, FC and unripened CM ghee made from DN cow milk and HF cow milk is given in Table 3, Table 4. There was no statistical difference in fatty acid profile between the different ghee samples on account of processing, including ripening across both milk sources (DN & HF). However, there are varying results on the effect of processing on fatty acid profile in the literature. While Tyagi et al. (2008) reported differences in C15:0, ω-6 and ω-3 fatty acids in fermented cream-butter ghee compared to unripened direct cream ghee, Bhide (2014) did not find differences in any individual fatty acids due to processing changes. Joshi (2014) reported a difference in only docosahexaenoic acid (DHA), one of the ω −3 fatty acids, and no difference in LA and ALA, nor the ratio of ω-3/ ω-6. Notably, even where the reported differences were statistically significant, they were small in absolute terms and unlikely to be relevant from a nutritional perspective. As an example, the increase in the DHA level of 0.083 ± 0.003 % in curd-butter ghee compared to 0.062 ± 0.002 % in direct cream ghee reported in Joshi (2014) was still too low compared to the World Health Organization (WHO) recommended 200–500 mg/day intake of DHA + EPA to prevent chronic diseases (World Health Organization, 2003). CLA is another class of fatty acids of interest in ghee. One of the most abundant forms of CLA in milk fat is cis-9, trans-11 octadecadienoic acid (Butler et al., 2009). In our study, the levels of cis-9, trans-11 octadecadienoic acid remained unchanged during the ripening process as CLA content in CD ghee and CM ghee did not show any variations. Literature remains divided as some studies have reported that CLA content in dairy products did not vary after processing and fermentation (Rodríguez-Alcalá & Fontecha, 2007; Shantha et al., 1996) while other studies have reported statistically significant variations (Kim & Liu, 2002; Rodríguez-Alcalá et al., 2014; Tyagi et al., 2008).

As mentioned in the introduction, the contradictions in the findings of the different studies are likely because of differences in processing conditions and analytical methods. Moreover, even where statistical differences have been reported in fatty acid profile on account of processing, these have been small in magnitude and unlikely to have nutritional significance.

#### 3.1.2 Comparison of fatty acids in ghee of DN and HF cows

A comparison of the fatty acid profile of DN and HF cow ghee did not show any difference under all three processes - CM, CD and FC at the chain length class level (*p* < 0.05) (Table 5). At the degree of saturation class level, while SFA and MUFA did not vary between the two cow breeds, significant differences were observed in the PUFA, with HF cow ghee having significantly higher PUFA levels than DN cow ghee for all three processes. HF ghee also showed significantly higher levels of ω-6 and ω-3 fatty acids, specifically ALA, than DN ghee for all three processes. However, the ratio of ω-3/ ω-6 in ghee was not different between the two breeds. Odd-chain fatty acid C15:0 and CLA were marginally higher in DN compared to HF in all three processes (Table 5).

**Table 5:** Comparison of fatty acids in HF and DN cow ghee processed with three methods (FA mg/g ghee)⁎⁎⁎

Fatty acid groups	DN Curd-butter	HF Curd-butter	DN Cream-butter	HF Cream-butter	DN Fermented Cream-butter	HF Fermented Cream-butter
Chain Length Classes						
SCFA	64.1 ± 0.88	64.8 ± 0.47	62.7 ± 1.78	70.1 ± 2.94	61.0 ± 1.32	66.8 ± 2.64
MCFA	79.5 ± 1.94	83.2 ± 1.61	75.6 ± 1.45	84.3 ± 3.53	76.7 ± 2.54	79.8 ± 3.43
LCFA	798 ± 2.4	794 ± 2.06	804 ± 3.44	787 ± 5.1	794 ± 2.06	795 ± 6.28
Degree of Saturation Classes						
SFA	667 ± 6.0	671 ± 3.69	679 ± 8.8	677 ± 4.31	671 ± 3.69	670.0 ± 3.0
MUFA	242 ± 5.09	233 ± 2.96	232 ± 7.13	227 ± 3.71	233 ± 2.96	234 ± 2.39
PUFA	32.4 ± 0.85^b^	37.7 ± 1.27^a^	30.9 ± 1.84	36.9 ± 1.01	30.5 ± 1.07^b^	37.5 ± 1.73^a^
Individual fatty acids						
Butyric acid (SCFA)	39.2 ± 0.81	40.7 ± 0.15	38.9 ± 1.18	44.2 ± 2.13	37.6 ± 0.85	42.2 ± 1.54
C15:0 (Odd-chain fatty acid)	10.8 ± 0.04	10.4 ± 0.74	11.3 ± 0.23	10.1 ± 0.44	11.0 ± 0.12	10.1 ± 0.51
α-Linolenic acid (ALA) ω-3	3.23 ± 0.08^b^	4.18 ± 0.19^a^	3.12 ± 0.18^b^	4.15 ± 0.29^a^	2.95 ± 0.15^b^	4.25 ± 0.11^a^
Conjugated fatty acid (CLA) (c-9, t-11)	8.81 ± 0.40	7.6 ± 0.25	8.5 ± 0.54	7.35 ± 0.23	8.61 ± 0.31	7.73 ± 0.35
Specialty groupings						
Odd-chain fatty acids (C11:0, C13:0, C15:0, C17:0, C17:1)	20.69 ± 0.29	20.01 ± 2.58	21.18 ± 0.3	19.67 ± 1.19	20.72 ± 0.16	19.42 ± 1.27
ω-3	3.23 ± 0.08^b^	4.18 ± 0.19^a^	3.12 ± 0.18^b^	4.15 ± 0.29^a^	2.95 ± 0.15^b^	4.25 ± 0.11^a^
ω-6	20.3 ± 0.56^b^	26.0 ± 1.37^a^	19.3 ± 1.16^b^	25.4 ± 1.2^a^	19 ± 0.82^b^	25.5 ± 1.69^a^
Ratio ω-6/ ω-3	6.28	6.22	6.18	6.12	6.44	6.0

Abbreviations DN=Deoni, HF=Holstein friesian, CD=Curd-butter ghee, CM = Cream-butter ghee, FC=Fermented cream-butter ghee.

SCFA = Short Chain Fatty Acids; MCFA = Medium Chain Fatty Acids; LCFA = Long Chain Fatty Acids; SFA = Saturated Fatty Acids; USFA = Unsaturated Fatty Acids; PUFA = Polyunsaturated Fatty Acids.

⁎ Values are mean ± SEM of triplicates of three samples.

⁎⁎ Different alphabets in a row between pairs depict statistical difference (p < 0.05).

### 3.2 Characterisation of ghee flavours

#### 3.2.1 Comparison of volatile compounds in ghee

The peaks obtained in the GC–MS were annotated using the National Institute of Standards and Technology (NIST) database and literature wherever a match percentage of 90 % or greater was found. The analysis of 6 samples, performed in duplicate, yielded a total of 57 peaks, of which 50 compounds were categorized into seven distinct functional groups. The remaining 7 compounds were not assigned to any specific functional group and placed under the ‘others’ group. These functional groups were acids (7), alcohols (4), aldehydes (11), alkanes (6), heat degradation products (HDP) (7), ketones (9) and lactones (6)**.** The number of compounds in the six ghee samples ranged from 34 to 47 (Table 6).

**Table 6:** Number of volatile compounds found in ghee and their mean peak area (×10^7^)⁎

Samples		Acids	Alcohols	Aldehydes	Alkanes	Heat Degradation Products	Ketones	Lactones	Others	Total compounds / area
DN Cream-butter	No of compounds	5	3	9	1	1	6	6	3	34
	Peak Area	1.71	0.33	0.48	0.04	0.28	1.65	2.12	0.22	6.83
DN Curd-butter	No of compounds	6	4	9	4	3	7	6	3	42
	Peak Area	6.12	0.45	1.2	0.09	0.83	1.54	2.77	0.41	13.41
DN Fermented cream-butter	No of compounds	7	3	10	3	3	7	6	4	43
	Peak Area	10.11	1.23	1.29	0.19	1.34	1.84	2.56	0.6	19.16
HF Cream-butter	No of compounds	6	4	8	2	7	9	6	5	47
	Peak Area	0.9	1.68	0.64	0.16	1.10	3.98	1.32	0.82	10.6
HF Curd-butter	No of compounds	7	4	10	2	4	8	6	5	46
	Peak Area	4.24	1.16	1.12	0.2	1.11	2.5	2.02	0.4	12.74
HF Fermented cream-butter	No of compounds	6	4	8	3	6	8	6	5	46
	Peak Area	4.1	1.38	1.11	0.24	1.58	1.69	1.5	1.07	12.67

DN=Deoni; HF = Holstein Friesian.

⁎ Values are mean of the triplicate analysis of a sample.

The number of compounds and their corresponding total peak area was higher in the two ripened ghee, CD and FC, compared to the unripened CM ghee (Table 6). This was true for both DN and HF cow milk. In particular, the acid group exhibited higher peak areas for the ripened ghee than the unripened CM ghee. Lactic acid was the most significant contributor to this increase (Table 7). It is formed due to the fermentation of milk and its significant contribution to the total peak area in the two ripened ghee samples (CD and FC) was expected. Hexanoic, octanoic and decanoic acids also have much higher peak areas in the ripened ghee samples than the unripened CM ghee. This is likely due to increased lipolysis of lipids resulting from the acidic conditions created during the ghee ripening process.

**Table 7:** Volatile compounds in ghee (Peak Area x10^7^) as impacted by cow breeds and process⁎

Sr. No.	Compound name⁎⁎a-c	Functional group	DN			HF		
			Curd-butter	Cream-butter	Fermented cream-butter	Curd-butter	Cream-butter	Fermented cream-butter
1	Acetic acid	Acids	0.24^b^	0.43^a^	0.21^bc^	0.11^bd^	0.065^d^	0.078^cd^
2	Butanoic acid		0.35	0.28	0.71	0.12	0.1	0.096
3	Decanoic acid		1.2	0.097	1.6	1.2	0.41	0.75
4	Hexanoic acid		0.5^ab^	0.59^ab^	0.95^a^	0.097^b^	0.093^b^	0.16^b^
5	Lactic acid		2.8^ab^	0^b^	4.9^a^	1.5^ab^	0.16^b^	2.7^ab^
6	Octanoic acid		1.1^a^	0.31^bc^	0.95^ab^	0.41^ac^	0.062^c^	0.29^bc^
7	Propanoic acid		–	–	0.79^a^	0.83^a^	–	–
8	2,3-Butanediol	Alcohols	0.099^ab^	–	0.76^ab^	0.67^ab^	0.083^ab^	0.92^a^
9	2-Propyl-1-pentanol		0.18	0.22	0.16	0.2	0.25	0.19
10	Ethanol		0.03^c^	0.06^bc^	–	0.06^bc^	0.08^ab^	0.10^a^
11	Furfuryl alcohol		0.14^b^	0.049^b^	0.3^b^	0.23^b^	1.3^a^	0.17^b^
12	2-Decenal	Aldehydes	0.02^ac^	0.02^bc^	0.04^ab^	0.04^ab^	0.04^a^	–
13	2-Nonenal		0.03^a^	0.03^ab^	0.03^a^	0.03^a^	0.01^c^	0.01^bc^
14	2-Octenal		–	0.04^c^	0.03^e^	0.03^d^	0.06^a^	0.05^b^
15	2-Undecenal		0.02^c^	–	0.05^a^	0.03^b^	–	–
16	Benzaldehyde		0.8 ^a^	0.08 ^c^	0.72 ^a^	0.70 ^a^	0.15^b^	0.61^a^
17	Decanal		0.02^d^	0.03^c^	0.04^b^	0.03^c^	0.05^a^	0.02^d^
18	Heptanal		0.06	0.04	0.07	0.05	0.05	0.04
19	Hexadecanal		–	0.03^b^	0.16^a^	0.04^b^	0.06^ab^	0.04^b^
20	Hexanal		0.05^a^	–	–	–	–	–
21	Nonanal		0.17	0.19	0.11	0.13	0.22	0.22
22	Octanal		0.03^b^	0.04^b^	0.05^ab^	0.05^ab^	–	0.11^a^
23	Dodecane	Alkanes	0.015^a^	–	–	–	–	–
24	Eicosane		0.019^b^	0.038^a^	–	–	–	–
25	Heptadecane		–	–	0.13^a^	0.097^c^	0.073^d^	0.13^b^
26	Hexacosane		0.033^a^	–	–	–	–	–
27	Hexadecane		–	–	0.029^d^	0.1^a^	0.086^b^	0.078^c^
28	Octadecane		0.026^b^	–	0.034^a^	–	–	0.026^b^
29	2(3H)-Furanone	HDP	0.087^ab^	–	0.056^b^	0.13^ab^	0.2^a^	0.092^ab^
30	2(5H)-Furanone		–	–	–	–	0.17^a^	0.046^b^
31	2,5-Dimethyl-4-hydroxy-3(2H)-furanone		–	–	–	–	0.27^a^	0.14^ab^
32	5-Hydroxymethyl-2[5H]-furanone		–	–	–	–	0.029^a^	–
33	5-Hydroxymethylfurfural		0.27^b^	–	0.86^a^	0.32^b^	0.038^b^	0.33^b^
34	Furfural		–	–	–	0.12^a^	0.18^a^	0.17^a^
35	Maltol		0.47	0.28	0.42	0.54	0.22	0.8
36	2-Heptanone	Ketones	0.1^ab^	0.21^ab^	0.16^ab^	0.13^ab^	0.29^a^	0.075^b^
37	2-Nonanone		0.2^b^	0.88^a^	0.21^b^	0.28^b^	0.65^ab^	0.18^b^
38	2-Pentadecanone		0.5	0.2	0.57	0.42	0.59	0.34
39	2-Pyrrolidinone		–	–	–	0.5	0.16	0.2
40	2-Tridecanone		0.34	0.15	0.36	0.51	0.79	0.38
41	2-Undecanone		0.33^b^	0.17^b^	0.37^ab^	0.53^ab^	0.95^a^	0.38^ab^
42	Butanone		–	–	–	–	0.051	–
43	Ethanone		0.022^c^	–	0.06^a^	0.037^b^	0.052^a^	0.034^b^
44	Furyl hydroxymethyl ketone		0.057^b^	0.052^b^	0.11^b^	0.085^b^	0.44^a^	0.092^b^
45	delta-Decalactone	Lactones	0.97^a^	0.7^bc^	0.79^ab^	0.62^bc^	0.49^c^	0.47^c^
46	delta-Dodecalactone		0.97^a^	0.76^ab^	0.98^a^	0.66^bc^	0.37^c^	0.5^bc^
47	delta-Hexalactone		0.057^ab^	0.1^a^	0.035^b^	0.033^b^	0.062^ab^	0.049^ab^
48	Delta-octalactone		0.18^ab^	0.19^a^	0.13^bc^	0.092^cd^	0.093^cd^	0.057^d^
49	delta-Tetradecalactone		0.13	0.052	0.091	0.18	0.046	0.064
50	gamma-Dodecalactone		0.46^ab^	0.32^bc^	0.54^a^	0.44^ab^	0.26^c^	0.37^bc^
51	2H-Pyran-2,6(3H)-dione	Others	–	–	–	0.015^b^	0.073^a^	0.027^b^
52	4H-Pyran-4-one		0.13	0.043	–	0.17	0.13	0.63
53	Azulene		0.11^b^	–	0.31^a^	–	–	–
54	Benzene		0.18	0.15	0.16	0.15	0.16	0.28
55	Caryophyllene		–	0.028^a^	0.037^a^	0.028^a^	–	0.035^a^
56	Hexadecene		–	–	0.096^a^	0.036^c^	0.075^b^	0.099^a^
57	Isomaltol		–	–	–	–	0.37	–

DN=Deoni; HF = Holstein Friesian.

⁎ Values are mean of the triplicate analysis of a sample.

⁎⁎ Only identified compounds are shown (−) = Not detected.

a-c Means in a row without a common superscript letter differ (*P* < 0.05) as analyzed by two-way ANOVA and the TUKEY test.

The methyl ketones, 2-heptanone and 2-nonanone were lower in the two CD and FC processes compared to the unripened CM ghee. The autooxidation of unsaturated lipids forms methyl ketones. Lower pH may decrease the rate of lipid oxidation due to the ease of proton transfer to alkyl (R·), alkoxy (RO·) and/or peroxyl lipid radicals (ROO·) formed during autoxidation (Grebenteuch et al., 2021). Since ripening leads to the generation of organic acids and reduced pH, it may explain the lower peak areas of 2-heptanone and 2-nonanone in the two ripened ghee samples.

Higher levels of lactones in ripened CD ghee compared to unripened CM ghee could also be attributed to the reduced pH resulting from ripening, as low pH favors the conversion of hydroxy acids to form cyclic esters or lactones (Sartori et al., 2021).

5-Hydroxymethylfurfural (5-HMF) and maltol, important volatile compounds associated with the flavour of ghee, also exhibited higher peak areas in the ripened ghee samples (CD and FC) compared to the unripened CM ghee. This is also expected, as both are thermally generated from sugars, and the rate of reaction increases in the acidic environment created by fermentation (Kavousi et al., 2015; Lewkowski, 2001). The above differences were largely consistent across the ghee made from the milk of the two cow breeds. This is expected, as the chemistry resulting in the generation of volatile compounds should be similar, regardless of the milk source.

Some variations in the volatile compounds were also observed between the two breeds. DN ghee had higher peak areas of acids and lactones compared to HF. In particular, the CD ghee of DN cow exhibited higher levels of lactones, especially, δ-decalactone, δ-dodecalactone and δ-octalactone, compared to CD ghee of HF cow.

**Fig. 1:**
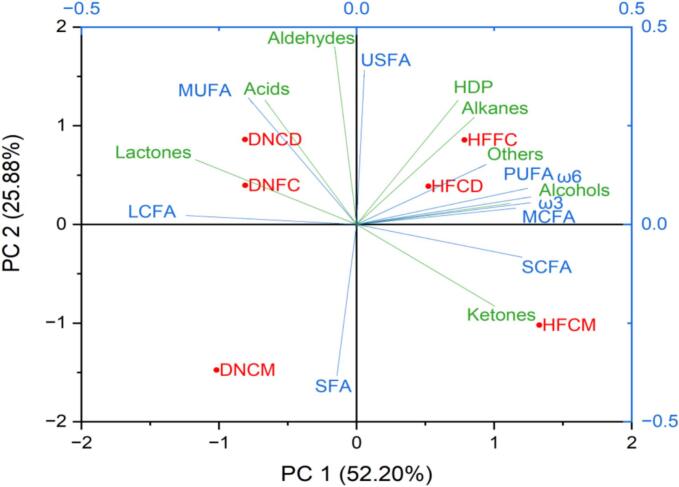
PCA illustrating the distribution of fatty acids and group of flavour compounds of clarified butter (ghee) based on processing methods and cow breeds.

The PCA plot indicates that processing methods, such as ripening, may significantly impact the volatile components of ghee than the cow breed (Fig. 1). Both cow breeds exhibit similar volatile profiles, as demonstrated by ripened ghee, suggesting uniformity in the flavour characteristics of the two ripening processes (CD and FC). Minor differences in fatty acid and volatile compounds of DN and HF cow ghee were observed in the PCA graph. CM ghee of DN and HF cows was characterized by fatty acid groups such as SFA, SCFA and flavour compounds of ketones group. In contrast, the CD ghee of DN cows was characterized by MUFA and LCFA, lactones, aldehydes, and acids. CD ghee of HF cow was recognized by unsaturated fatty acids like PUFA, ω-6, ω-3 and MCFA, along with flavour compounds of HDP, alcohols, alkanes, and others.

#### 3.2.2 Quantification of volatile compounds in ghee

Nine compounds with the highest peak areas were selected for quantification (Table 8). These included two thermally generated volatile compounds (5-HMF and maltol), three ketones (2-pentadecanone, 2-tridecanone and2-undecanone), four lactones (δ-octalactone, δ-decalactone, γ-dodecalactone and δ-dodecalactone).

**Table 8:** Quantified concentrations (μg/g) of nine flavour compounds in ghee⁎⁎⁎, a-c

Compound name	DN			HF		
	Curd-butter	Cream-butter	Fermented cream-butter	Curd-butter	Cream-butter	Fermented cream-butter
5-HMF	6.66 ± 1.06^b^	–	14.40 ± 0.90^a^	7.76 ± 2.83^b^	0.874 ± 0.43^b^	8.13 ± 4.89^b^
Maltol	12.1 ± 2.19	7.29 ± 1.97	10.7 ± 1.75	13.4 ± 3.45	5.25 ± 0.24	15.4 ± 6.03
2-Pentadecanone	0.63 ± 0.03	0.247 ± 0.03	0.69 ± 0.08	0.499 ± 0.17	0.707 ± 0.29	0.401 ± 0.16
2-Tridecanone	0.708 ± 0.03	0.381 ± 0.02	0.56 ± 0.02	0.734 ± 0.28	1.21 ± 0.46	0.517 ± 0.23
2-Undecanone	5.54 ± 0.58^ab^	2.74 ± 0.11^b^	5.82 ± 0.72^ab^	8.23 ± 2.49^ab^	14.8 ± 3.55^a^	5.76 ± 2.26^ab^
delta-Decalactone	2.48 ± 0.04^a^	1.81 ± 0.12^ab^	1.44 ± 0.48^ab^	1.4 ± 0.03^b^	1.06 ± 0.19^b^	1.01 ± 0.15^b^
delta-Dodecalactone	2.27 ± 0.13^a^	1.73 ± 0.03^ab^	2.18 ± 0.22^a^	1.41 ± 0.24^bc^	0.765 ± 0.08^c^	1.05 ± 0.07^bc^
Delta-octalactone	1.21 ± 0.02^a^	1.3 ± 0.005^a^	0.659 ± 0.06^b^	0.334 ± 0.07^c^	0.341 ± 0.12^c^	0.129 ± 0.04^c^
gamma-Dodecalactone	0.76 ± 0.042^a^	0.49 ± 0.08^ab^	0.762 ± 0.10^a^	0.548 ± 0.04^ab^	0.289 ± 0.07^b^	0.445 ± 0.06^ab^

nd = Not detected.

Abbreviations DN=Deoni, HF=Holstein Friesian, 5-HMF = 5-Hydroxymethylfurfural.

⁎ Values are mean ± SEM of the three triplicates of a sample.

⁎⁎ Only identified compounds are shown.

a-c Means in a row without a common superscript letter differ (*P* < 0.05) as analyzed by two-way ANOVA and the TUKEY test.

As also indicated in Table 7, a higher level of 5-HMF was found in ripened ghee, CD and FC ghee of both cow breeds (*p* < 0.05). 5-HMF was negligible in the unripened CM ghee of the two breeds. 5-HMF is a heat-generated flavour compound having caramel and sweet flavour notes. Higher levels of 5-HMF in ripened ghee can be attributed to the low pH produced during bacterial fermentation. In the Maillard reaction, low pH favors 1,2-enolization of Amadori products, resulting in the formation of 5-HMF and furfural. Whereas, at high pH, 2,3-embolization predominates to produce furanone (Boekel, 2006; Newton et al., 2012).

Maltol has a caramel-butterscotch-like flavour and is a primary Maillard reaction product generated by heat and caramelization reactions in ghee (Andrewes, 2012; Brien, 2009; Wadodkar et al., 2002). In the present study, we found that maltol in ghee remained unaffected by the processing methods in both breeds. Though not statistically significant, ripened CD and FC ghee of DN and HF breeds had higher levels of maltol than unripened CM ghee. With concentrations up to 15.4 ± 6.03 μg/g, maltol was the most abundant compound in the FC ghee of HF cow. The results align with past findings, indicating that maltol and 5-HMF are unique to traditional ghee processes involving ripening and were absent in commercial samples and butter oil (Wadodkar et al., 2002).

All the three ketones viz. 2-pentadecanone, 2-tridecanone and 2-undecanone were not affected by the ghee processing method in both breeds. Though in this study, ketones were found in smaller amounts than other flavour compounds, they play an important role in ghee flavour. The volatile compound 2-pentadecanone provides a fatty, floral and spicy flavour, while 2-tridecanone has a warm, oily, and herbaceous odor and 2-undecanone contributes a rue, fresh, green, and orange flavour (Erfani et al., 2019). The formation of methyl ketones in ghee occurs through the oxidation of lipids during heat processing, which produces carbonyl compounds, including methyl ketones, through hydrolysis and decarboxylation reactions (Grebenteuch et al., 2021; McSweeney & Sousa, 2000).

All the lactones, including δ-decalactone δ-dodecalactone, δ-octalactone and γ-dodecalactone were not affected by the process within the breed. However, there were variations between the processes across the two breeds as ripened CD ghee of DN cow had significantly higher levels of δ-decalactone compared to unripened CM ghee of HF cow. (Table 8). Though not statistically different, the amounts of δ-dodecalactone and γ-dodecalactone were higher in the ripened ghee (CD and FC) of both the cow breeds compared to the respective unripened CM ghee. Lactones contribute to the flavour of ghee by providing coconut, peach, sweet and fruity aromas and are produced through the lactonization of gamma and delta hydroxy fatty acid triglycerides (Chen et al., 2021). Because lactones have low flavour threshold values (FTV), they significantly enhance the flavour of ghee even at low concentrations. In a mixture, the FTV of δ-octalactone was 0.54, whereas the FTV of δ-decalactone and δ-dodecalactone were 2.7 and 5.4, respectively (Siek et al., 1970). In the present study, the high amounts of lactones in native DN cow ghee indicated that it is more flavourful than HF cow ghee. Also, higher lactone content in ripened ghee (CD and FC) of the two breeds suggests that fermentation plays a crucial role in the formation of more volatile flavour compounds in ghee. Lactones may also be formed during ripening with the involvement of microorganisms, such as yeasts and bacteria, which convert keto acids into hydroxy acids to form lactones (Vagenas & Roussis, 2012).

Comparing the two breeds, 5-HMF was significantly higher in FC ghee of DN cow compared to that of HF cow. Maltol and the two ketones (2-pentadecanone and 2-tridecanone) were not affected by the breeds. 2-undecanone in ghee was also affected by the breed as HF cow ghee samples showed higher levels of 2-undecanone than DN cow ghee, while DN cow ghee had significantly higher levels of all four lactones, i.e., δ-decalactone, δ-dodecalactone, δ-octalactone, and γ-dodecalactone, compared to HF cow ghee.

Ghee is a heated milk product that is characterized by distinct flavours produced as a result of heating. Flavour compounds in ghee, such as free fatty acids, lactones and carbonyls, result from the Maillard reaction, sugar dehydration and degradation (Shahidi & Hossain, 2022). Thermal decomposition of amino acids, lactose proteins, and fat also leads to the formation of these flavour compounds (Parliment et al., 1966; Regado et al., 2007). Free fatty acids generated through lipolysis during fermentation may play a crucial role in the flavour development in fat-rich dairy products (Forss, 1979; McSweeney & Sousa, 2000). Factors that affect the flavour formation in ghee include clarification temperature, ripening, nature of the starter culture and storage (Cadwallader & Singh, 2014; Suwarat & Tungjaroenchai, 2013). Studies in the literature indicated the role of fermentation in producing higher concentrations of flavour compounds in traditional ripened ghee. Higher total carbonyls were also produced in ripened ghee, contributing to higher flavour scores than unripened ghee, without adding new fatty acids (Yadav & Srinivasan, 1985). This probably explains the results of the present study, as significant variations were not found in the fatty acid composition of ghee due to ripening, whereas considerable differences were observed in the flavour profile of ghee made with ripened processes (CD and FC) compared to unripened CM ghee. Joseph & Appachar (1980) also indicated that metabolic byproducts from the starter culture *Streptococcus lactis* ssp. *diacetylactis* likely contributes to the high proportions of volatile compounds**.** Ripening cream with *Streptococcus lactis* subsp. *diacetylactis* also shows enhanced flavour generation in ghee (Joseph & Srinivasan, 1980). While lactones, free fatty acids, and carbonyl compounds were the key components contributing to the ghee flavour, it was challenging to introduce a ghee-like flavour in dairy products (Debnath & Ghosh, 2018). This was likely because not all of the ghee's components had been identified or their precise amounts were unknown. This study extends previous findings by quantifying specific flavour compounds, such as ketones, maltol, lactones, and 5-HMF in ghee produced from two milk sources of DN and HF cow breeds using three processing methods.

The volatile flavour compounds in CD and FC ghee of both DN and HF cows were similar, indicating that the FC process can be a scalable alternative to the traditional CD process for the production of ghee with a comparable flavour profile. The fermentation process and cow breed both significantly affect flavour molecules in ghee, highlighting the complexity of flavour formation. This study reinforces established dairy flavour chemistry paradigms and expands them with a detailed quantitative analysis of key flavour compounds, offering insights into optimizing ghee flavour in commercial production. Consistent with prior research, the study found increased lactones and maltol in ripened ghee, suggesting that fermentation plays a crucial role in shaping the flavour profile of traditional ghee.

## 4 Conclusion

The present work studied the effect of three different processes (CD, CM, and FC) on the fatty acid composition profile and volatile profile of ghee. The consistency of the effect of processing was checked by conducting the evaluation across two sources of milk – a native DN and a crossbred HF. A total of 26 fatty acids and 57 volatile compounds were identified and compared across the resulting six ghee samples.

No statistical difference was observed in the individual 26 fatty acid profiles or at the class levels of fatty acids between the ghee made from the three processing methods in either of the two milk sources. Comparing the fatty acid composition of similarly processed ghee from the two cow breeds revealed a difference in the PUFA fraction, with both ω-3 and ω-6 fatty acids being higher in HF. The opposite was observed for CLA and C15:0, where levels were marginally higher for DN across all three processes.

Unlike for fatty acids, differences were observed in volatile compounds resulting from processing methods. The two ripened ghee samples, CD and FC ghee had similar profiles to each other but were distinct from the unripened CM ghee. Ripened ghee, CD, and FC had more acids, alcohols, HDP, lactones, ketones, 5-HMF, and maltol than unripened CM ghee. These trends were consistent across DN and HF cow milk sources. The lower pH from ripening likely drives these volatile profile differences, warranting further pH-based studies.

The results indicate that processing methods can alter volatile compounds without significantly affecting fatty acids in ghee. This paper has studied only 26 fatty acids. Nearly 400 fatty acids have been reported in milk fat (MacGibbon, 2020). Ghee also has phospholipids, sphingolipids, and traces of bioactive peptides. Further investigation of this wider set of molecules may reveal differences resulting from processing, some of which could be physiologically significant. Untargeted lipidomics and metabolomics studies of ghee are approaches that can shed further light on the effect of processing ghee on the broader set of molecules.

## CRediT authorship contribution statement

**Deepshikha Kataria:** Writing – original draft, Software, Methodology, Investigation, Formal analysis, Conceptualization. **Gurmeet Singh:** Writing – review & editing, Supervision, Resources, Project administration, Funding acquisition.

## Declaration of competing interest

The authors declare that they have no known competing financial interests or personal relationships that could have appeared to influence the work reported in this paper.

## Data Availability

Data will be made available on request.

## Appendix A Supplementary data

Supplementary material 1 Image 1

Supplementary material 2 Image 2
