# Supplementary material for: Effect of processing methods on fatty acid composition and flavour profile of clarified butter (ghee) obtained from Deoni and Holstein Friesian cow breeds
Source: Food Chem X. 2025 Apr 22;27:102489. doi: 10.1016/j.fochx.2025.102489 (PMC12131252; doi:10.1016/j.fochx.2025.102489)
Supplement: Supplementary file 1 — Supplementary material 1 [file mmc1.docx]

**Table S1: Processing methods studied in the literature for their effects on fatty acids and volatile compounds in ghee**

| **Reference*** | **Parameter Studied** | **Curd-butter ghee** | **Cream-butter ghee** | **Fermented cream-butter ghee** | **Fermented cream ghee** | **Direct cream ghee** | **Unspecified Industrial Ghee** | **Butter Oil** |
| --- | --- | --- | --- | --- | --- | --- | --- | --- |
| **Yadav and Srinivasan (1984)** | Fatty acids | x |  |  | x | x |  |  |
| **Tyagi et al. (2008)** | Fatty acids |  |  | x |  | x |  |  |
| **Joshi (2014)** | Fatty acids and volatile compounds | x |  |  |  | x |  |  |
| **Bhide (2014)** | Fatty acids  And volatile compounds | x |  | x |  | x |  |  |
| **Joseph and Appachar (1980)** | Aroma volatiles |  |  |  | x | x |  |  |
| **Yadav & Srinivasan (1985)** | Aroma volatiles |  |  |  | x | x |  |  |
| **Wadodkar et al (2002)** | Aroma Volatiles | x |  |  |  |  | x | x |

*Note: Only those studies have been included in which more than one processing methods were studied and fatty acid profile and/or aroma volatile profile were compared

x-denotes process studied in respective paper

**Table S2: Literature on the effect of processing methods on the fatty acid composition of ghee**

| **Reference** | **Processes Compared** | **Source of milk used for making ghee (cow breed)** | **Bacterial strain used for ripening** | **Significant results on fatty acid composition** |
| --- | --- | --- | --- | --- |
| **Joshi (2014)** | Curd-butter ghee vs Direct cream ghee | Not mentioned | Curd culture  (specifications not given) | DHA higher in curd-butter ghee |
| **Bhide (2014)** | Curd-butter ghee vs fermented cream-butter ghee vs direct cream ghee | Not mentioned | Mixed curd culture of *Streptococcus thermophilus, Lactobacillus delbrueckii subsp. Bulgaricus, Lactobacillus lactis subsp. Lactis biovar. Diacetylactis* | No differences were found in fatty acids due to ripening. |
| **Tyagi et al. (2008)** | Fermented cream-butter ghee vs direct cream ghee | Crossbreed (Holstein Friesian x Tharparkar) | *Lactococcus lactis sp. Lactis* | CLA, ω-3 and ω-6 were higher in fermented cream-butter ghee  No difference in SFA, MUFA & PUFA  No difference in SCFA, MCFA |
| **Yadav and Srinivasan (1985)** | Curd-butter ghee, fermented cream ghee vs direct cream ghee | Not mentioned | *Streptococcus lactls sub sp. diacelyiactis* | Fermented cream ghee differed quantitatively from the direct cream ghee (control) and was  comparable to curd-butter ghee. Free fatty acids, total carbonyls and flavor score were higher in fermented cream ghee. No new fatty acids were found due to fermentation. |

**Table S3: Standard curve equations of the nine volatile compound standards used in the study**

| **S.No.** | **Compounds** | **RT** | **Linear equation** | **r^2^** | **Linearity range (µg/ml)** |
| --- | --- | --- | --- | --- | --- |
| 1 | Maltol | 15.16 | y = 3.9417x - 0.9753 | 0.9852 | 12.0-2.0 |
| 2 | 5-Hydroxymethylfurfural | 19.24 | y = 4.0388x + 0.304 | 0.9913 | 12.0-2.0 |
| 3 | Undecanone | 19.57 | y = 6.2819x - 1.7435 | 0.9954 | 3.0-0.25 |
| 4 | δ-Octalactone | 21.25 | y = 17.325x - 3.4224 | 0.9754 | 3.0-0.25 |
| 5 | 2-Tridecanone | 25.12 | y = 58.84x - 7.8676 | 0.9828 | 3.0-0.25 |
| 6 | δ-Decalactone | 26.67 | y = 41.128x - 4.8558 | 0.9921 | 3.0-0.25 |
| 7 | 2-Pentadecanone | 29.54 | y = 81.798x - 1.6093 | 0.9962 | 3.0-0.25 |
| 8 | γ-Dodecalactone | 30.32 | y = 68.532x - 6.0627 | 0.989 | 3.0-0.25 |
| 9 | δ-Dodecalactone | 31.10 | y = 44.421x - 3.5003 | 0.9812 | 3.0-0.25 |
