# Supplementary material for: Effect of processing methods on fatty acid composition and flavour profile of clarified butter (ghee) obtained from Deoni and Holstein Friesian cow breeds
Source: Food Chem X. 2025 Apr 22;27:102489. doi: 10.1016/j.fochx.2025.102489 (PMC12131252; doi:10.1016/j.fochx.2025.102489)
Supplement: Supplementary file 2 — Supplementary material 2 [file mmc2.docx]

Supplementary Material-2


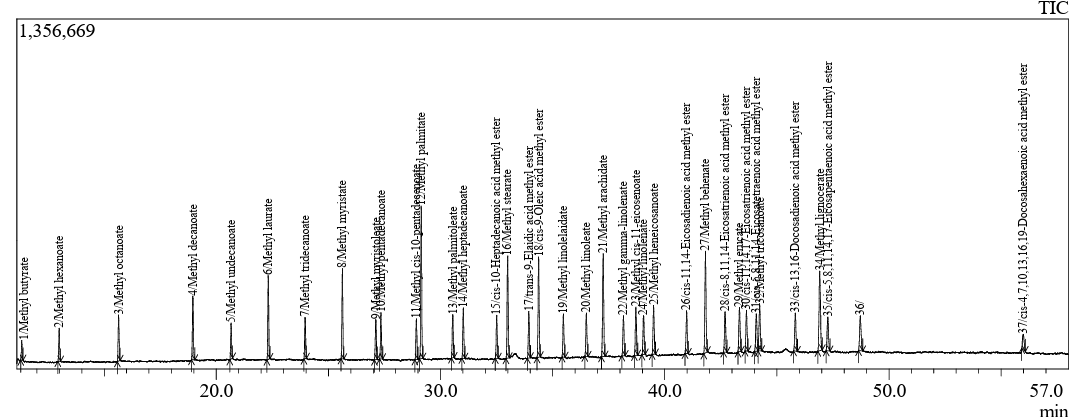


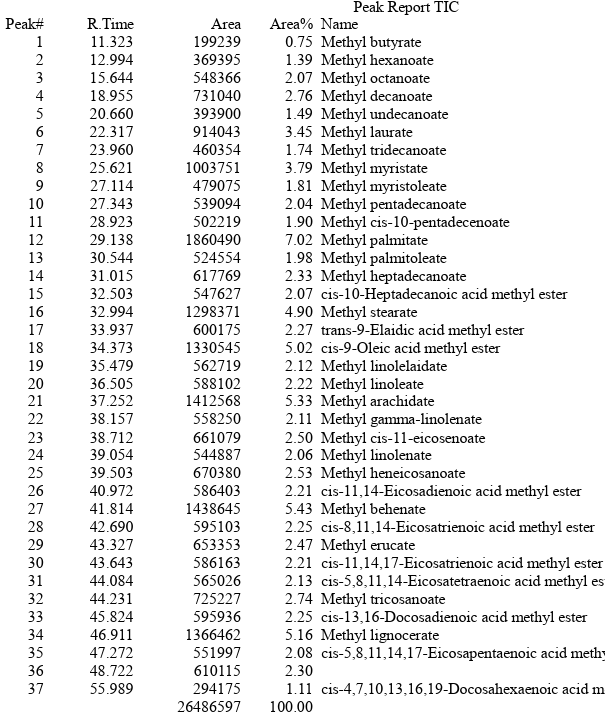


Figure S1: Chromatogram and peak table of the fatty acid standard (37 FAMEs mix)


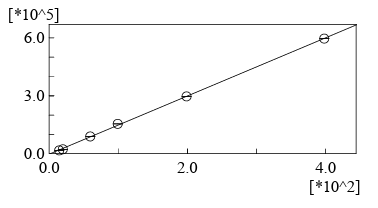

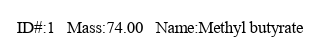

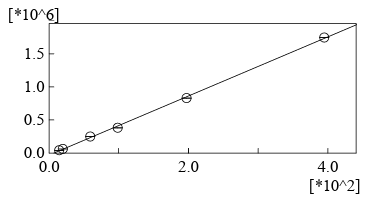

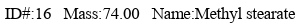

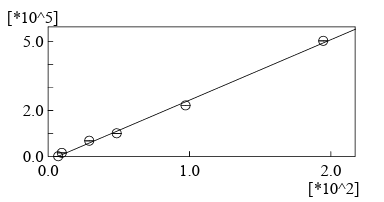

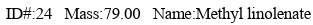

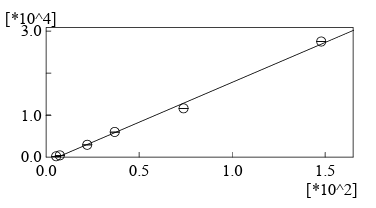

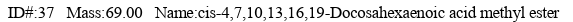


Figure S2: Standard curves for selected Fatty Acid Methyl Esters (from 37 FAMEs standard)


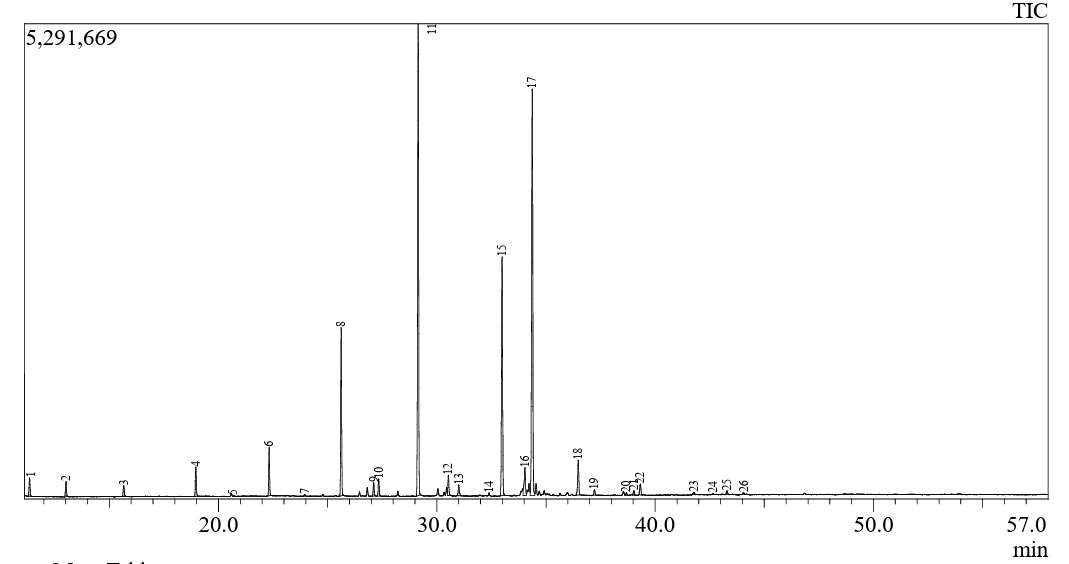


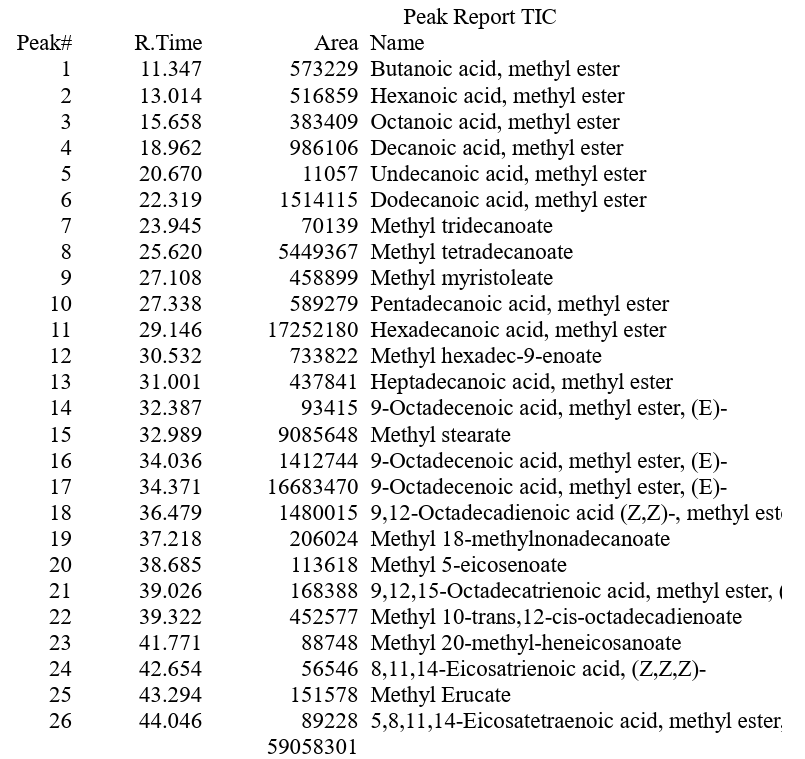


Figure S3: Chromatogram and peak table (TIC) of the fatty acid methyl esters (FAMEs) of ghee sample

Linear curves, chromatograms and mass spectra of the nine volatile compounds


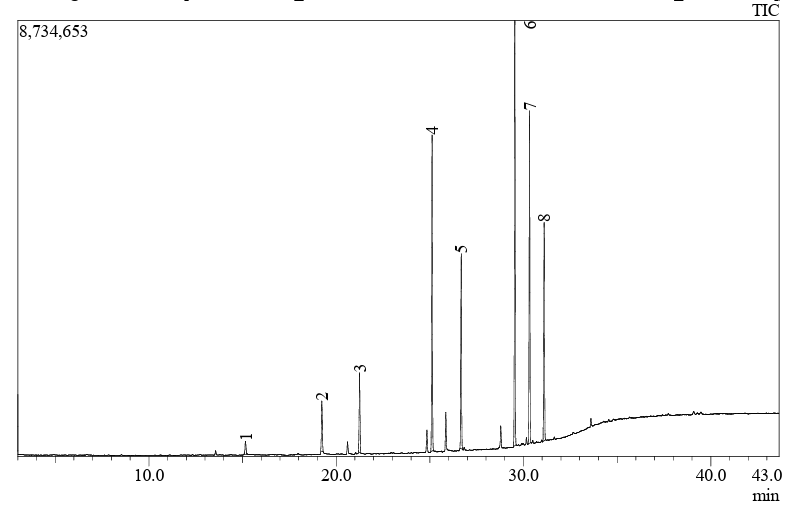


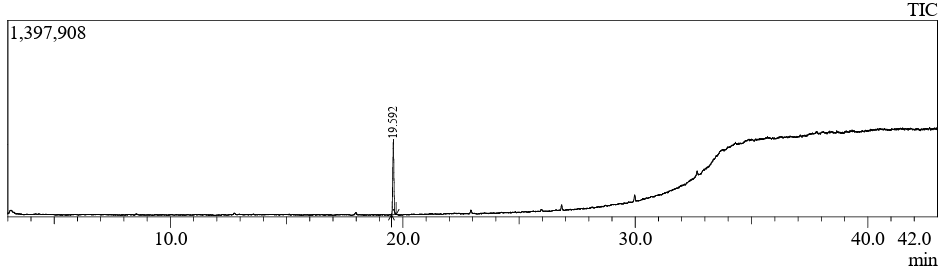

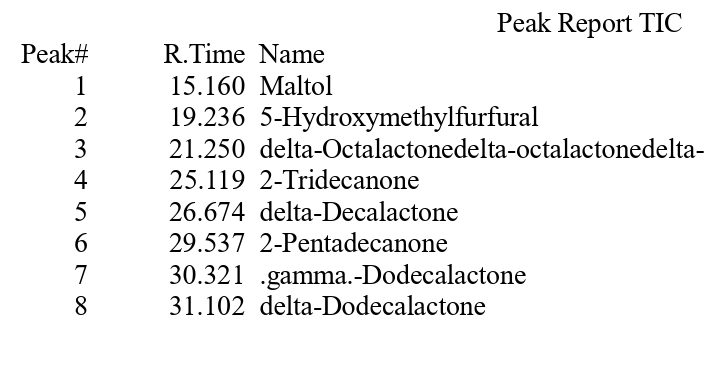


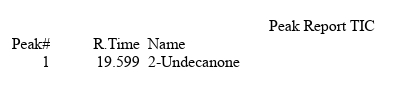


S4: Chromatograms of the nine flavor compounds analyzed in the study

Linear curves of the nine volatile compounds


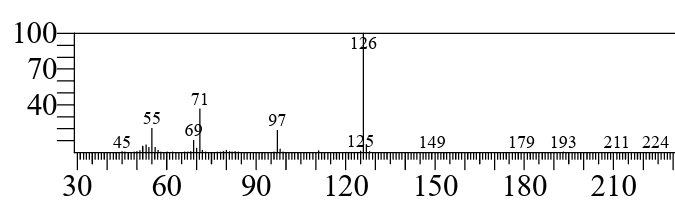

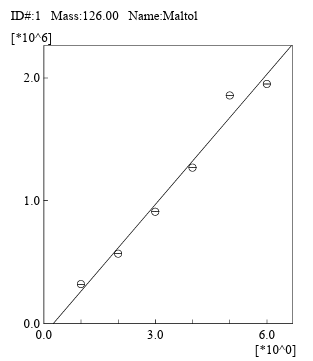


Figure S5: Calibration curve and mass spectra of maltol


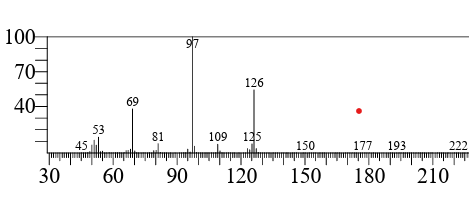

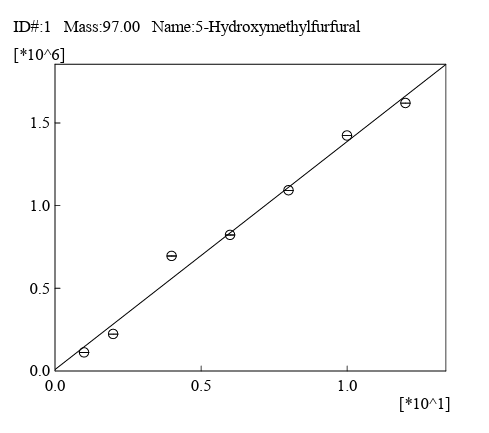


Figure S6: Calibration curve and mass spectra of 5-Hydroxymethylfurfural


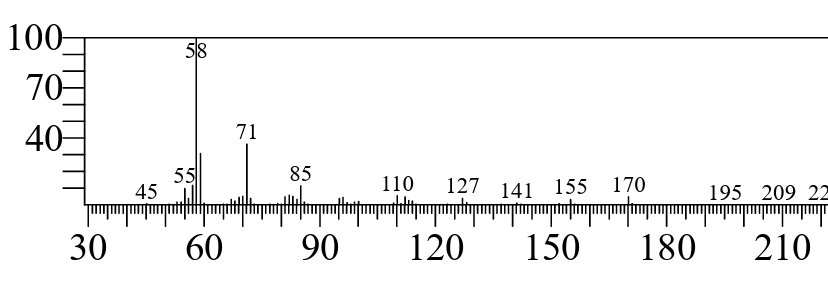

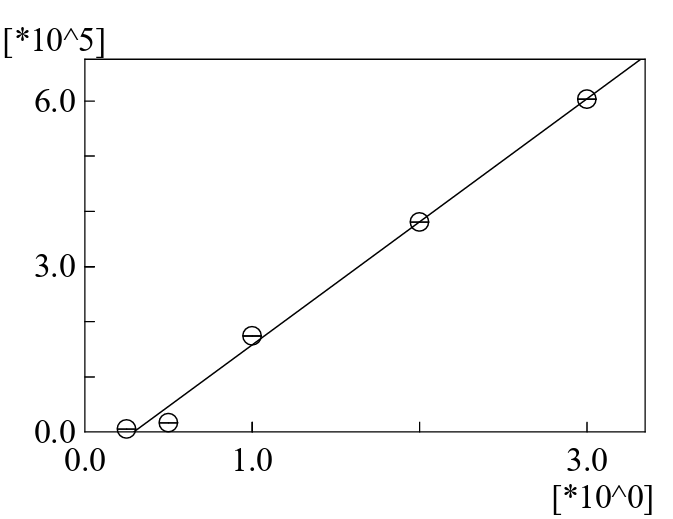

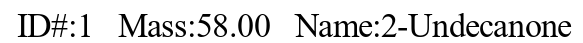


Figure S7: Calibration curve and mass spectra of 2-Undecanone


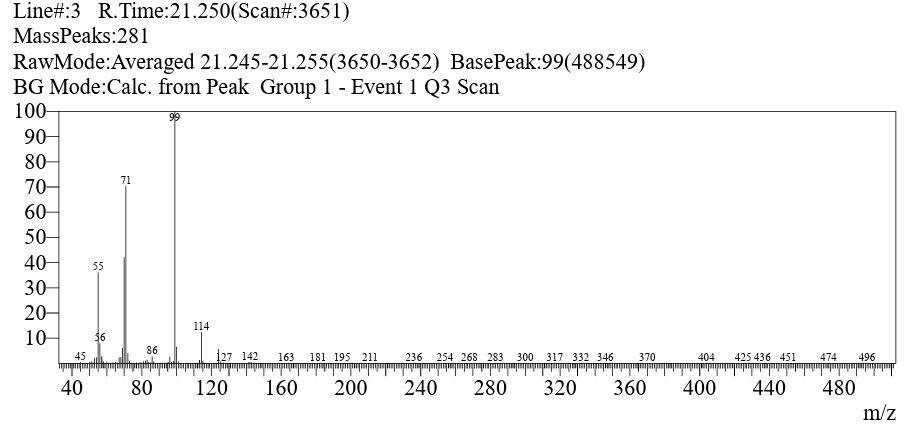

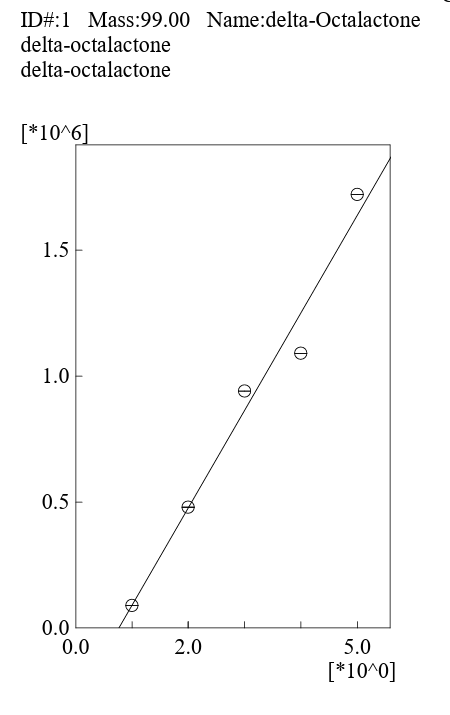


Figure S8: Calibration curve and mass spectra of δ-Octalactone


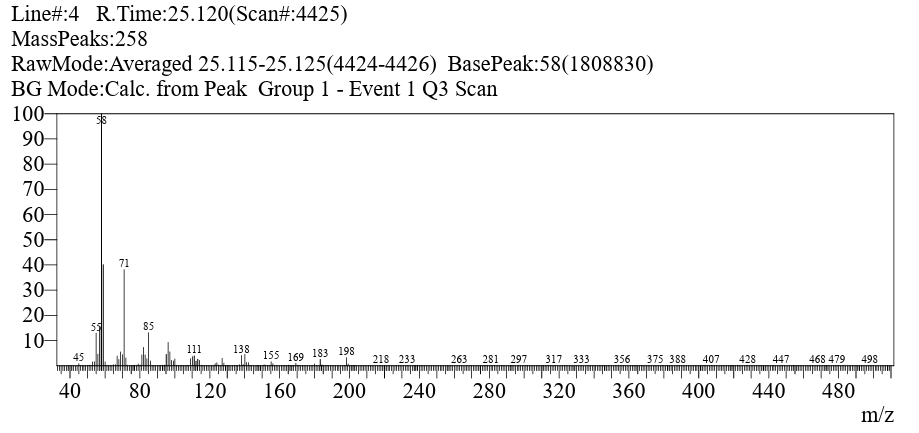

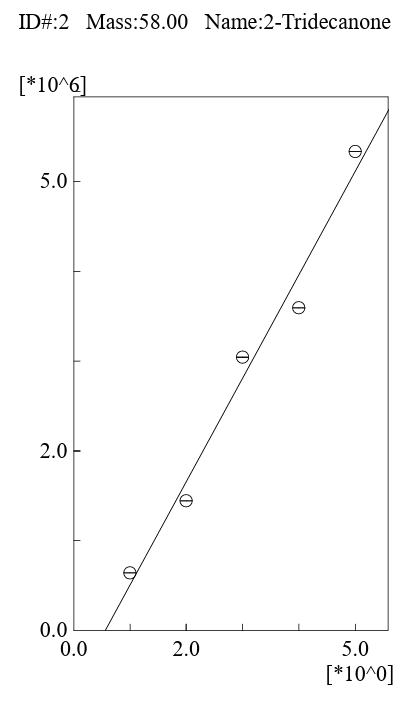


Figure S9: Calibration curve and mass spectra of 2-Tridecanone


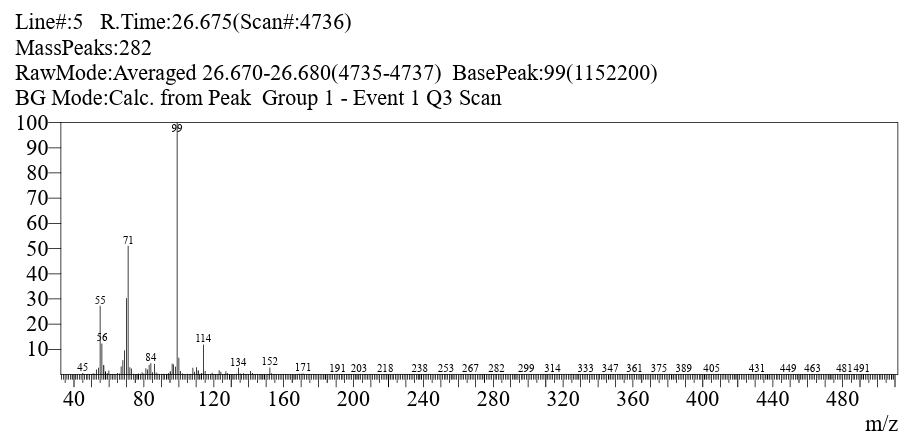

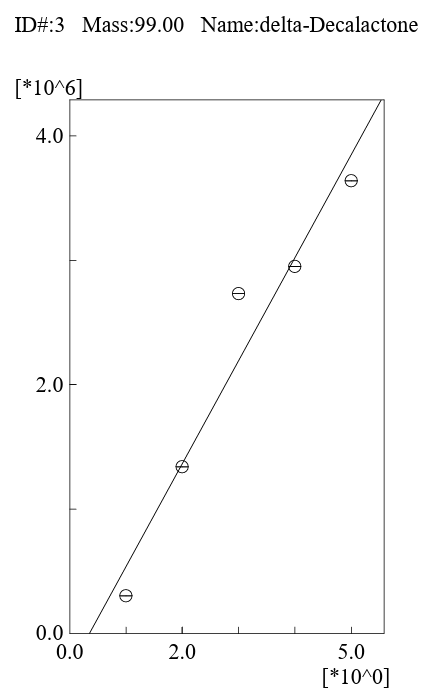


Figure S10: Calibration curve and mass spectra of δ-decalactone


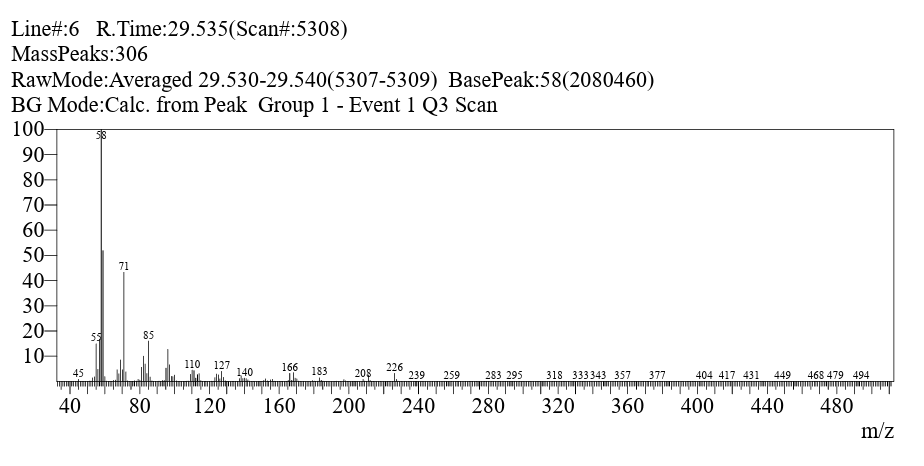

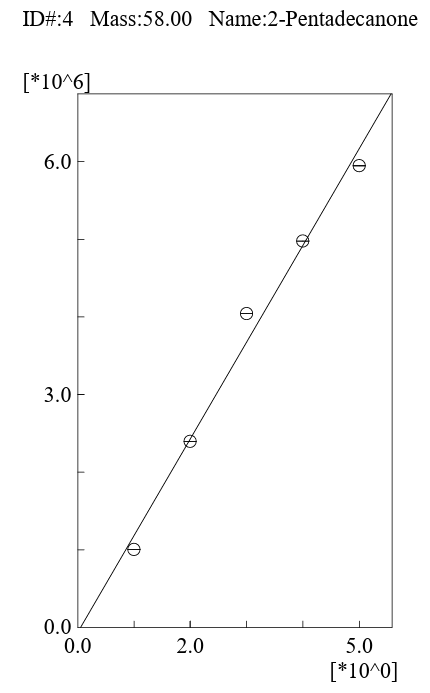


Figure S11: Calibration curve and mass spectra of 2-Pentadecanone


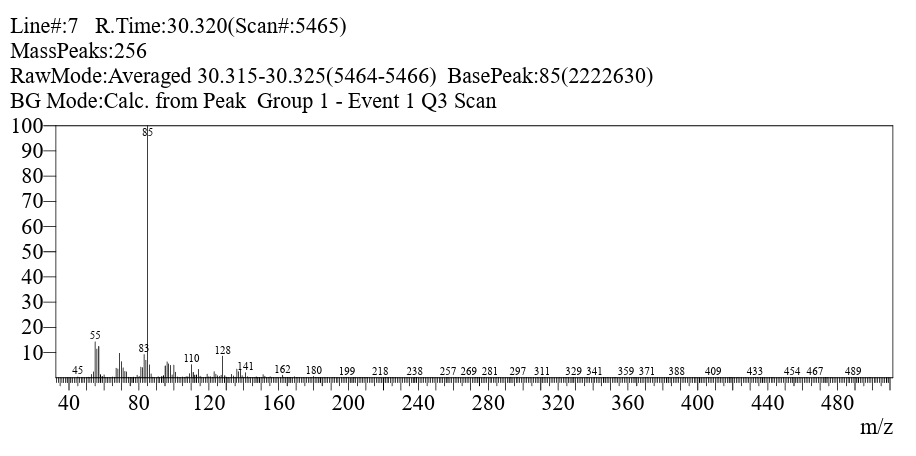

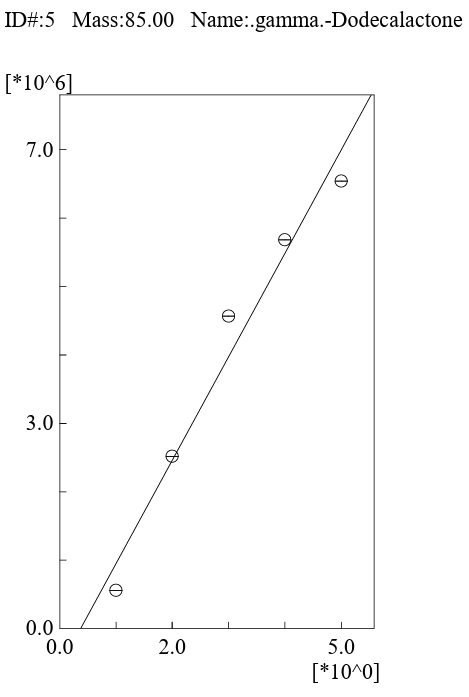


Figure S12: Calibration curve and mass spectra of γ-dodecalactone


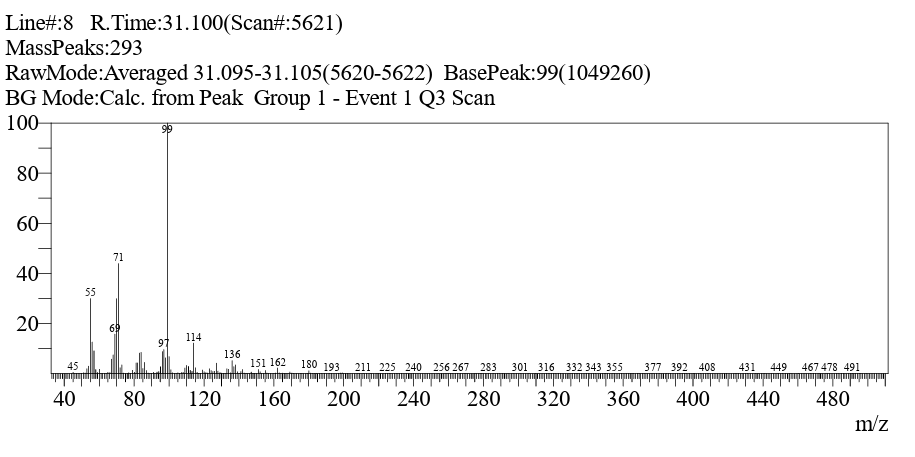

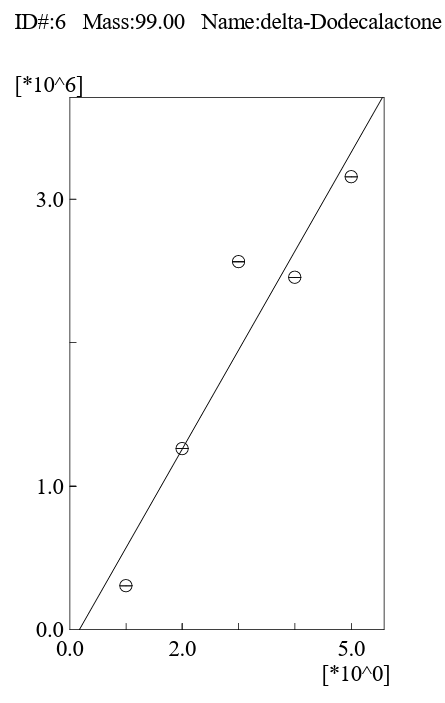


Figure S13: Calibration curve and mass spectra of δ-dodecalactone


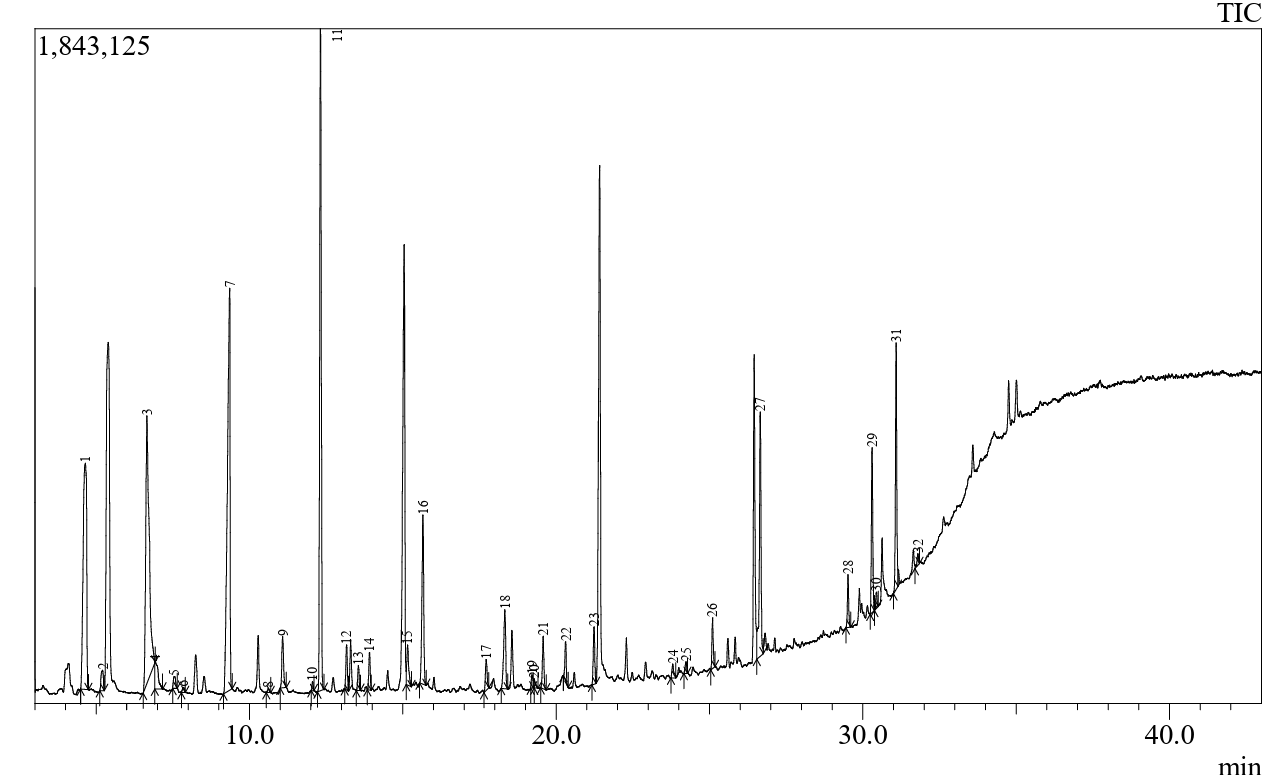


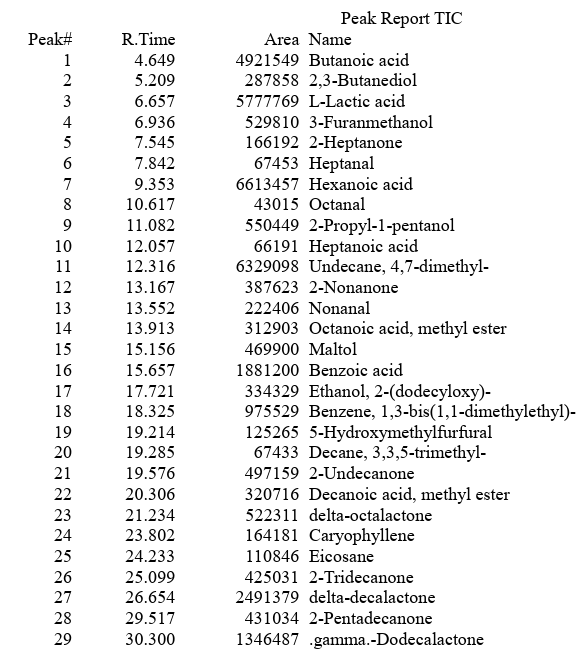

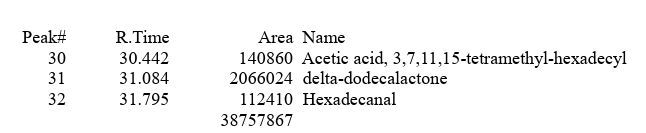


Figure S14: Chromatogram and peak table (TIC) of the volatile compound analysis of ghee sample
